# Beyond the Colours: Discovering Hidden Diversity in the Nymphalidae of the Yucatan Peninsula in Mexico through DNA Barcoding

**DOI:** 10.1371/journal.pone.0027776

**Published:** 2011-11-23

**Authors:** Blanca R. Prado, Carmen Pozo, Martha Valdez-Moreno, Paul D. N. Hebert

**Affiliations:** 1 El Colegio de la Frontera Sur, Unidad Chetumal, Quintana Roo, Mexico; 2 Biodiversity Institute of Ontario, University of Guelph, Ontario, Canada; University of Guelph, Canada

## Abstract

**Background:**

Recent studies have demonstrated the utility of DNA barcoding in the discovery of overlooked species and in the connection of immature and adult stages. In this study, we use DNA barcoding to examine diversity patterns in 121 species of Nymphalidae from the Yucatan Peninsula in Mexico. Our results suggest the presence of cryptic species in 8 of these 121 taxa. As well, the reference database derived from the analysis of adult specimens allowed the identification of nymphalid caterpillars providing new details on host plant use.

**Methodology/Principal Findings:**

We gathered DNA barcode sequences from 857 adult Nymphalidae representing 121 different species. This total includes four species (*Adelpha iphiclus*, *Adelpha malea*, *Hamadryas iphtime* and *Taygetis laches*) that were initially overlooked because of their close morphological similarity to other species. The barcode results showed that each of the 121 species possessed a diagnostic array of barcode sequences. In addition, there was evidence of cryptic taxa; seven species included two barcode clusters showing more than 2% sequence divergence while one species included three clusters. All 71 nymphalid caterpillars were identified to a species level by their sequence congruence to adult sequences. These caterpillars represented 16 species, and included *Hamadryas julitta*, an endemic species from the Yucatan Peninsula whose larval stages and host plant (*Dalechampia schottii,* also endemic to the Yucatan Peninsula) were previously unknown.

**Conclusions/Significance:**

This investigation has revealed overlooked species in a well-studied museum collection of nymphalid butterflies and suggests that there is a substantial incidence of cryptic species that await full characterization. The utility of barcoding in the rapid identification of caterpillars also promises to accelerate the assembly of information on life histories, a particularly important advance for hyperdiverse tropical insect assemblages.

## Introduction

The Order Lepidoptera includes about 160,000 described species of butterflies and moths [1]–[3], and it is thought that a similar number await discovery [3], [4]. For example, Lamas [5] noted that collections hold numerous undescribed species and concluded that many more species await discovery in the Neotropics. One third of all butterfly species belong to the Nymphalidae [6], a family that occurs in all faunal regions, but is most diverse in the Neotropics [1]. Approximately 570 species of Nymphalidae have been reported from Mexico, representing 28% of its butterfly fauna [4]. About one quarter of this total (121 species) occur in the Yucatan Peninsula (Campeche, Quintana Roo and Yucatan States).

Although the Nymphalidae has been widely studied, gaps remain in knowledge of their systematics [7], [8], life cycles and host plants. There are some identification keys for species of economic importance [9], [10], but most larval stages remain difficult to assign to a species. Rearing caterpillars is the traditional way to connect larval and adult stages, and the success of this approach has been shown in Costa Rica where Janzen and Hallwachs have now reared 4,500 species, nearly half of the local fauna [11]–[14]. However, this approach takes much time, staff and substantial funding [14].

DNA barcoding, the sequence analysis of a short standard segment of the cytochrome *c* oxidase subunit I gene (COI) provides a rapid way to probe biodiversity [15], [16]. It also makes it possible to identify any life stage by matching barcode records from unknown caterpillars with a barcode library constructed through the analysis of adult specimens as evidenced by work on the moth *Exoteleia dodecella* [17] and a study on the identification of host caterpillars to track host-parasitoids interactions [18]. DNA barcoding works particularly well for Lepidoptera and some past and recent studies have shown that it aids the discovery of new species, especially cryptic taxa [19]–[28]. Based on these results, we employed DNA barcoding to extend understanding of the immature stages of the Nymphalidae from the Yucatan Peninsula and the incidence of cryptic species. By providing access to new information on adult and caterpillar morphology, our work will aid better understanding of the systematics of Nymphalidae.

In this study, COI sequences were obtained from 121 species of adult Nymphalidae from the Yucatan Peninsula held in El Colegio de la Frontera Sur-Chetumal (ECOSUR) collection and previously identified through morphological study. The barcode analysis of these species revealed both four overlooked species in the collection (known species that were misidentified) and provided evidence for several cryptic species (undescribed new species). As well, the reference sequence library created through the analysis of adults was a useful tool to identify caterpillars.

## Results

**Figure 1:**
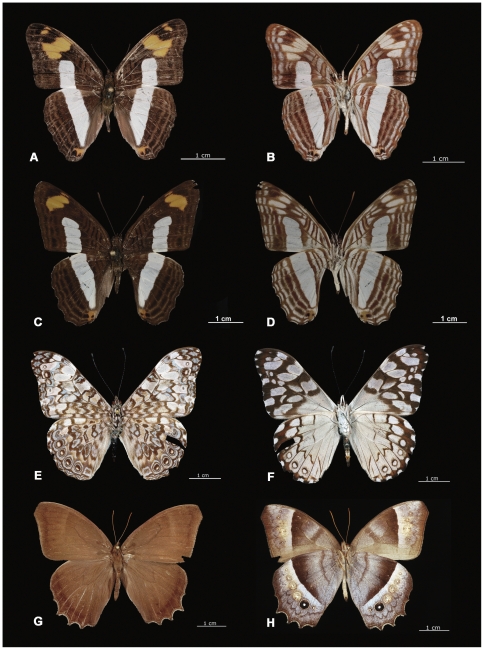
Four overlooked Nymphalidae species within the Lepidoptera collection of ECOSUR from the Yucatan Peninsula. A–B) *Adelpha malea*; C–D) *Adelpha iphiclus*; E–F) *Hamadryas iphtime*; G–H) *Taygetis laches*. Each species is shown in dorsal and ventral view. *A. iphiclus* is a new record for the Yucatan Peninsula while *T. laches* is a new record for Mexico. Photos by Humberto Bahena.

**Figure 2:**
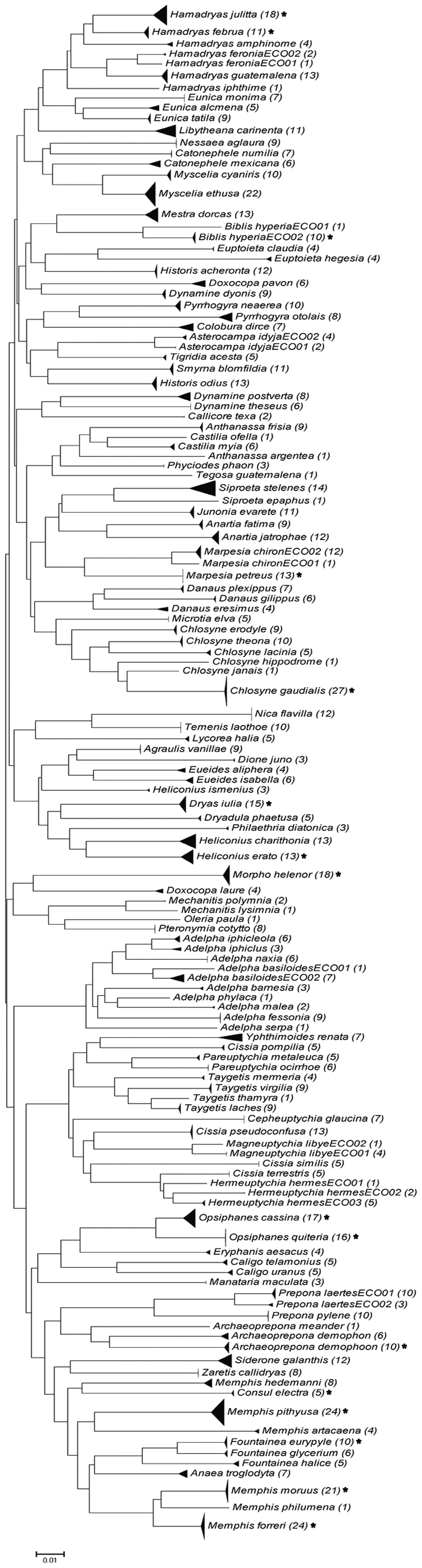
Neighbour Joining tree for 121 species of Nymphalidae from the Yucatan Peninsula. Tree is based on genetic distances (K2P) for the barcode region of the COI gene; the height of each triangle indicates mean intraspecific sequence divergence, while the base of the triangle provides a rough indication of the number of specimens analyzed. Brackets enclose the number of barcodes generated for each species and those with an asterisk include both caterpillars and adults.

We obtained sequences from 857 adult specimens and 71 caterpillars. Most (95%) of these sequences were longer than 600 bp, 3.4% ranged from 400–599 bp and 1.6% varied between 267–399 bp. The coupling of barcode results with subsequent morphological analysis revealed four species that had been overlooked in ECOSUR collection, *Adelpha iphiclus, Adelpha malea, Hamadryas iphtime* and *Taygetis laches* (Figure 1). The first of these species represents a new record for the Yucatan Peninsula, while the last is a new record for Mexico. Before barcoding these specimens were mistaken with closely morphologically similar taxa (*A. iphicleola/A. nea, A. barnesia*, *H. feronia* and *T. thamyra* respectively). In two other cases, the barcode results provoked the merger of specimens initially assigned to different species. Specimens originally identified as *Opsihanes tamarindi* and *O. quiteria* showed no barcode divergence and morphological re-examination indicated that all were actually *O. quiteria*. Similarly, specimens identified as *Junonia evarete* and *J. coenia* showed no barcode divergence and morphological re-analysis indicated that all specimens were actually *J. evarete*. The NJ tree for the 121 Nymphalidae species (Figure 2) shows that each of the species possessed a diagnostic array of barcode sequences.

**Figure 3:**
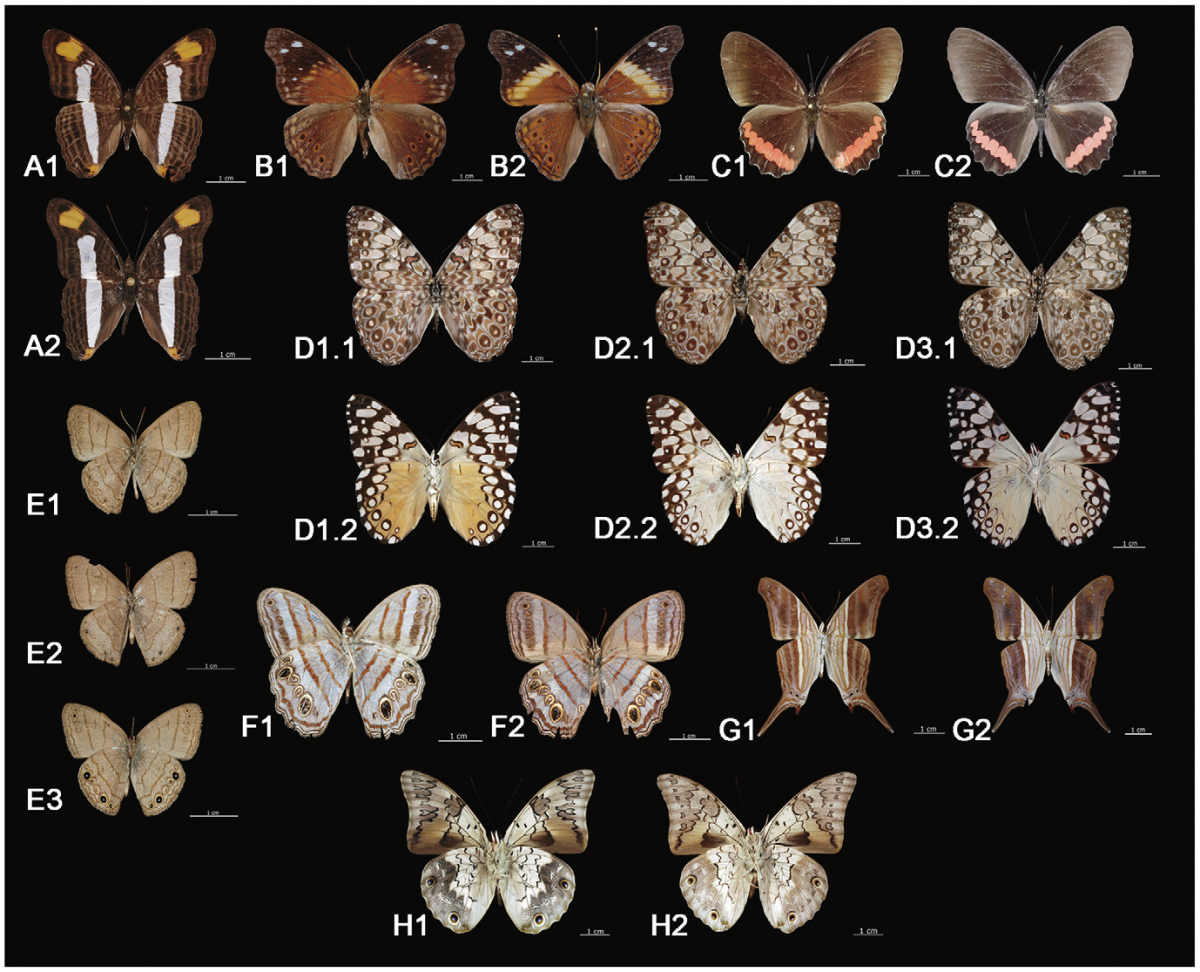
Species of Nymphalidae from the Yucatan Peninsula showing deep sequence divergence (>2%) at COI. Sequences in 7 species were partitioned into two clusters, while one species included three clusters. A1) *Adelpha* basiloidesECO01, A2) *A.* basiloidesECO02; B1) *Asterocampa* idyjaECO01, B2) *A.* idyjaECO02; C1) *Biblis* hyperiaECO01, C2) *B.* hyperiaECO02; D1) *Hamadryas* feroniaECO01, D2) and D3) *H.* feroniaECO02, images D1.1, D2.1 and D3.1 showing dorsal view; E1) *Hermeuptychia* hermesECO01, E2) *H.* hermesECO02, E3) *H.* hermesECO03; F1) *Magneuptychia* libyeECO01, F2) *M.* libyeECO02; G1) *Marpesia* chironECO01, G2) *M.* chironECO02; H1) *Prepona* laertesECO1 and H2) *P.* laertesECO02. Images A, B, C and E are dorsal views while F, G and H are ventral views. Photos by Humberto Bahena.

Eight of the 121 species included two or three barcode clusters and we assigned an interim name to each cluster (Table 1, Figure 3). We did not detect any striking differences in adult morphology between the members of the different clusters in most of these species, but we did not examine genitalia. However, there were exceptions. The two clusters of *Asterocampa idyja* showed clear morphological divergence (Figure 3, B1 and B2) which has in the past been thought to reflect melanic versus normal forms in Mexico [29]. The dorsal wing surfaces of *Hamadryas feronia* showed no obvious difference (Figure 3, D1.1, D2.1 and D3.1), but the ventral surfaces (Figure 3, D1.2, D2.2 and D3.2) showed variation that has, in the past, been viewed as seasonal or regional variation [30]. Although more detailed morphological, ecological and genetic studies need to be carried out to decide the status of the barcode clusters in these eight species, we expect that many reflect new taxa or subspecies that should be raised to species status and that were previously unknown from Mexico. After excluding these eight species, members of the remaining 113 species possessed a mean intra-specific distance of 0.27% and maximum of 1.90% (Table 2). Divergences among congeneric species were considerably higher, averaging 7.95%.

**Table 1:** Eight species of Nymphalidae from the Yucatan Peninsula which include two or three clusters of barcode sequences showing >2% sequence divergence.

Species	Interim name	Mean Dist(%)	BOLD ID engine (public)	Sim(%)
*Adelpha basiloides*	*A.* basiloidesECO01	3.15%	*Adelpha* basiloidesDHJ01	99.7
	*A.* basiloidesECO02		*Adelpha* basiloidesDHJ02	99.6
*Asterocampa idyja*	*A.* idyjaECO01	2.24%	*Asterocampa idyja*	99.9
	*A.* idyjaECO02		Not match found	
*Biblis hyperia*	*B.* hyperiaECO01	4.67%	*Biblis* hyperiaDHJ02	99.3
	*B.* hyperiaECO02		*Biblis* hyperiaDHJ01	100
*Hamadryas feronia*	*H.* feroniaECO01	2.01%	*Hamadryas* guatemalenaDHJ02	100
	*H.* feroniaECO02		*Hamadryas farinulenta*	99.8
*Hermeuptychia hermes*	*H.* hermesECO01	3.64%	Not match found	
	*H.* hermesECO02		Not match found	
	*H.* hermesECO03		Not match found	
*Magneuptychia libye*	*M.* libyeECO01	2.32%	Not match found	
	*M.* libyeECO02		*Magneuptychia libye*	99.7
*Marpesia chiron*	*M.* chironECO01	2.10%	*Marpesia Chiron*	99.4
	*M.* chironECO02		*Marpesia chiron*	99.8
*Prepona laertes*	*P.* laertesECO01	2.82%	*P. laertes Octavia*	98.2
	*P.* laertesECO02		Not match found	

Also is indicated the identification through the BOLD ID engine with the percentage of similarity.

**Table 2:** Genetic distances (K2P) for the barcode region of the COI gene for 113 species of Nymphalidae from the Yucatan Peninsula.

	Comparisons	Min Dist(%)	Mean Dist(%)	Max Dist(%)	SE Dist(%)
Within Species	4334	0	0.27	1.90	0.005
Within Genus	6605	2.10	7.95	13.33	0.029
Within Family	332267	6.88	13.53	21.04	0.003

**Figure 4:**
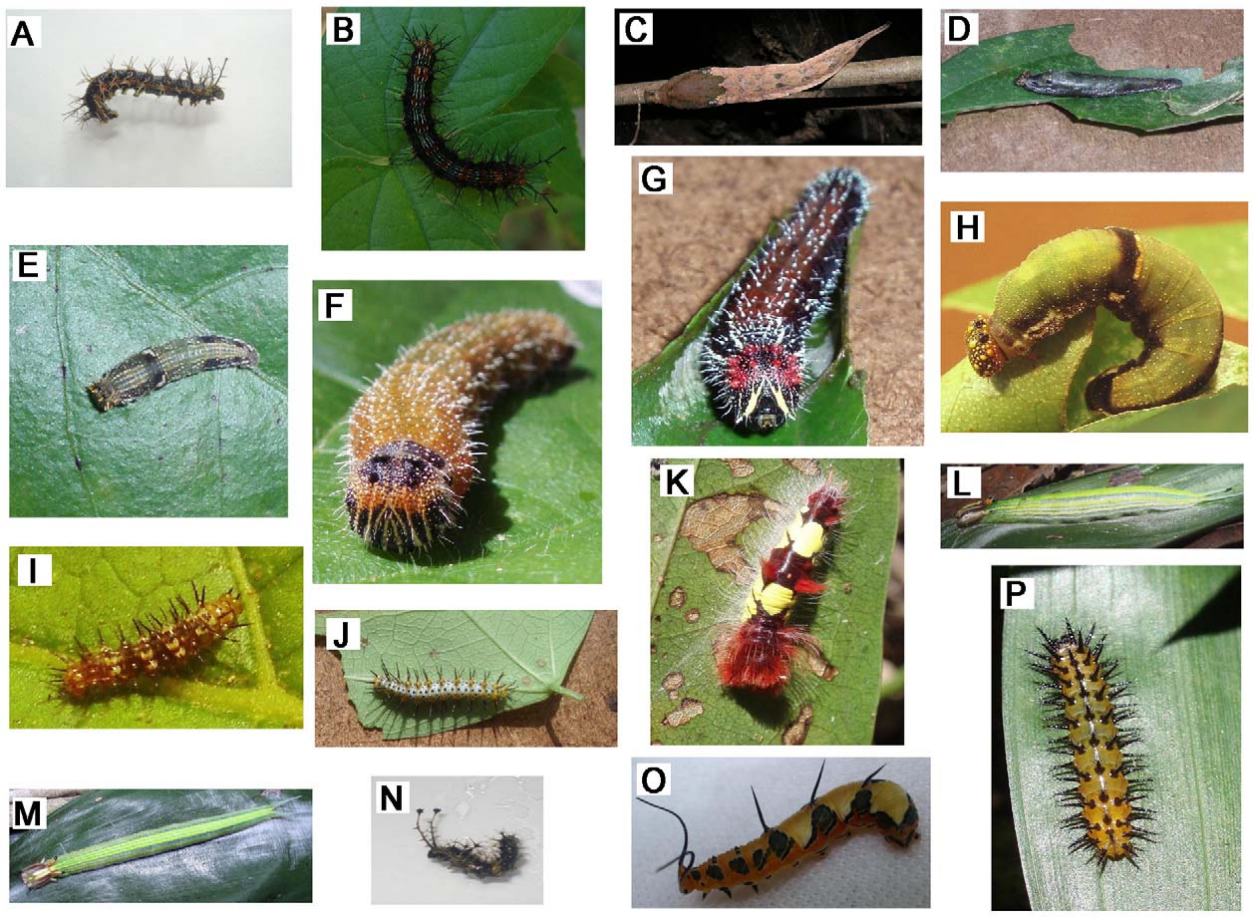
Caterpillars for 16 species identified by matching sequences with the adults DNA barcode reference library. A) *Hamadryas februa*, B) endemic *H. julitta*, C) *Archaeoprepona demophoon*, D) *Consul electra*, E) *Fountainea eurypyle*, F) *Memphis forreri*, G) *M. moruus* H) *M. pithyusa*, I) *Dryas iulia*, J) *Heliconius erato*, K) *Morpho helenor*, L) *Opsiphanes cassina*, M) *O. quiteria*, N) *Biblis* hyperiaECO02, O) *Marpesia petreus* and P) *Chlosyne gaudialis*. Images A and N are from specimens in alcohol. All photos by Tijl Essens and Blanca R. Prado.

Barcode analysis enabled the identification of all 71 nymphalid caterpillars collected in a field survey, assigning them to 16 different species (Figure 4) as they showed less than 2% K2P divergence from adults sequenced in this study (Table 3).

**Table 3:** Species assignments for caterpillars showing that the closest sequence match with adults of Nymphalidae was always much less than 2% (K2P).

Caterpillar species	Mean Dist(%)
*Archaeoprepona demophoon*	0.04
*Biblis* hyperiaECO02	0.17
*Chlosyne gaudialis*	0.22
*Consul electra*	0.04
*Dryas iulia*	0.64
*Fountainea eurypyle*	0.02
*Hamadryas februa*	0.32
*Hamadryas julitta*	0.81
*Heliconius erato*	0.40
*Marpesia petreus*	0.46
*Memphis forreri*	0.18
*Memphis moruus*	0.06
*Memphis pithyusa*	0.58
*Morpho helenor*	0.35
*Opsiphanes cassina*	0.86
*Opsiphanes quiteria*	0.02

## Discussion

### Adult identification

Our study has examined DNA barcode variation in all 121 species of Nymphalidae known from the Yucatan Peninsula. Our results established that most of these species (109 of 121) possess little sequence variation at COI, but revealed 12 taxa with lineages showing more than 2% divergence. Four of these cases had a simple explanation. They reflected described species (*Adelpha malea*, *Adelpha iphiclus*, *Hamadryas iphthime* and *T, laches*) whose presence had been overlooked because of their morphological similarity to other species. Although *A. malea* (Figure 1, A–B) can only be distinguished from *A. barnesia* by careful observation of venation and colour pattern, the two taxa have a barcode divergence of 7.46%. *A. barnesia* occurs throughout the Atlantic and Pacific mid-south of Mexico including the Yucatan Peninsula [31], [32]. *A. malea* has been reported from the Yucatan Peninsula [33], but Mexican collections include only one specimen from the west (Veracruz State) [31]. Our analysis reveals more specimens that have been overlooked and we expect that many individuals of this species are currently misplaced in collections as *A. barnesia*.

*Adelpha iphiclus* (Figure 1, C–D) and *A. iphicleola* are sister taxa whose morphological separation is extremely difficult (the orange subapical mark is slightly wider in *A. iphicleola* than in *A. iphiclus).* The two species show a mean sequence divergence of just 2.32%, suggesting that their phenotypic similarity reflects a recent evolutionary origin. Nevertheless, these species occur sympatrically through much of Central America [33]. *A. iphicleola* occurs widely in Mexico including the Yucatan Peninsula, while *A. iphiclus* was previously known from the western side of the country [31], [33]. As Willmott reports *A. iphiclus* from Belize [33], our detection of its presence in the Yucatan Peninsula is not unexpected. Our work revealed one additional specimen that was originally identified as *A. nea*, but that grouped with *A. iphiclus* in the NJ tree and subsequent morphological analysis indicated that it was the latter taxon.

**Figure 5:**
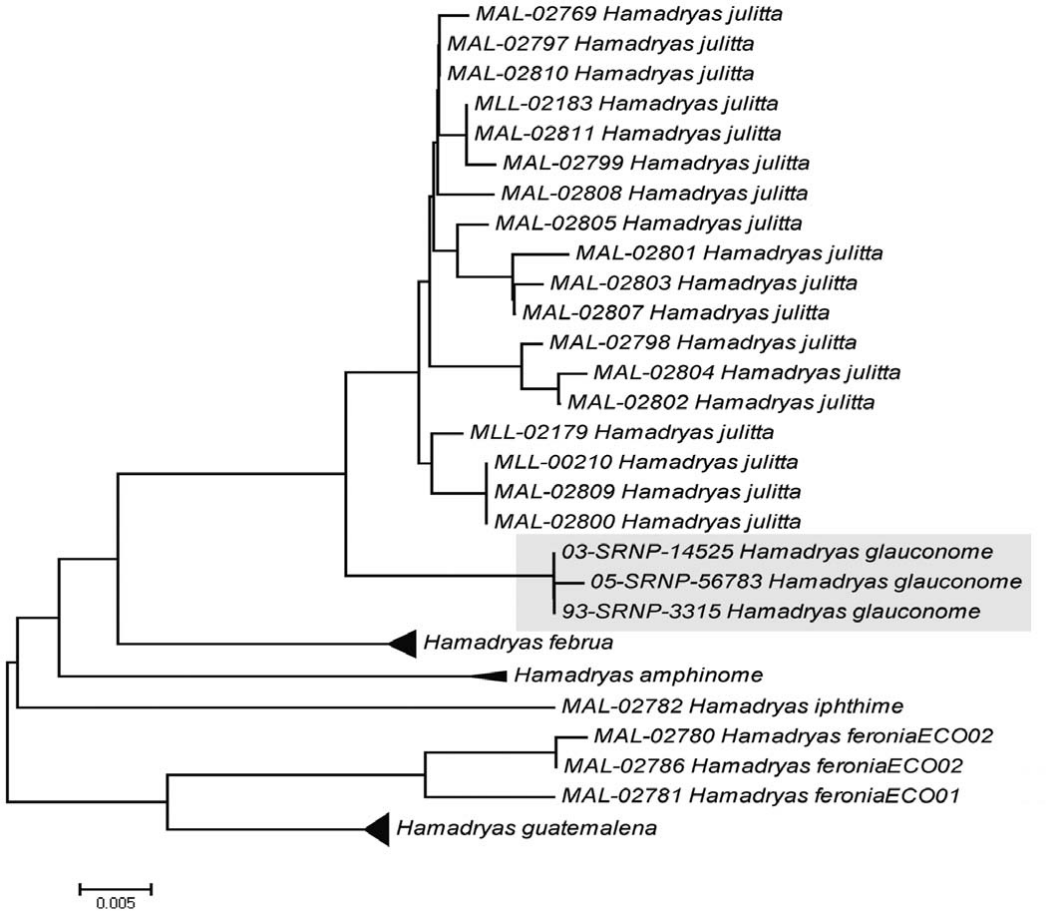
Neighbor-joining tree for the genus *Hamadryas*. *H. julitta,* an endemic from the Yucatan Peninsula, is most closely related to *H. glauconome* from Costa Rica (in grey). Barcoding also revealed *H. iphthime*, a species overlooked in the collection because of its close similarity to *Hamadryas feronia*, and two clusters of *H. feronia.*

Species in the genus *Hamadryas* are often difficult to discriminate because different species show similar colours and patterns. Moreover, intraspecific variation is considerable and some variants were described as species, but have now been relegated to synonymy [5], [30]. The most careful study on *Hamadryas* reduced the genus from 92 taxa to 41 (20 species and 21 subspecies) [30]. In Mexico 10 species are recognized from this genus [31]. Four species (*H. februa*, *H. feronia*, *H. guatemalena* and *H. amphinome)* with broad distributions in Mexico have been reported from the Yucatan Peninsula together with the endemic *H. julitta,* [31], [32]. Our analyses revealed that specimens morphologically identified as *H. feronia* fell into two clusters showing 7.28% sequence divergence, and subsequent morphological analysis revealed that *H. iphthime* had been overlooked by our morphological studies (Figures 1 E–F, 2 and 5). This species has been reported from north of Quintana Roo [30], but no prior Mexican collection contains *H. iphthime* from the Yucatan Peninsula. There remained three specimens morphologically identified as *H. feronia*, but they split into two clusters whose members show slight differences in colour (Figure 3, D). The subspecies *H. feronia farinulenta* [31], [32] is known from Mexico and the ID engine on BOLD showed that the *H.* feroniaECO02 has 99.8% similarity with *H. farinulenta*. Interestingly, our,*H.* feroniaECO01 matches with *Hamadryas* guatemalenaDHJ02 (See Table S1 for information on specimens compared from the public database). In the past *H. guatemalena* and *H. feronia* were mistaken because of their morphological similarity [30], but *Hamadryas guatemalena* in this study is well separated from *H. feronia* (Figure 5). According to Jenkins description of *H. feronia farinulenta*, it is possible that *H.* feroniaECO01 is actually the “real” *H. feronia farinulenta* and that *H.* feroniaECO02 is the subspecies described as *Ageronia feronia nobilita* by Fruhstofer, but relegated as a variation of *H. feronia farinulenta* and later synonymized by Jenkins [30]. Although it is likely that they represent different species, we have employed interim names (*H.* feroniaECO01 and *H.* feroniaECO02) until their taxonomic status is investigated in more detail.

Another interesting species was the endemic *H. julitta* ( = *honorina*) that is very morphologically similar to *H. glauconome* (not reported in the Peninsula). In fact, previous investigators have considered *H. julitta* as a subspecies of *H. glauconome*, but there are significant genitalic differences between the two species [30]. Our results support this conclusion as *H. julitta* showed 2% divergence from *H. glauconome* (Figure 5). Although the barcodes of these two species only differed by 8 diagnostic nucleotide positions, other studies have revealed cases where different species (e.g. Hesperiidae species of *Polyctor*, *Neoxeniades* and *Cobalus*) show only 1–3 diagnostic substitutions in the barcode region [24]. No standard sequence threshold separates species and the discrimination of closely allied species must often rely on joint information from genetics, morphology, ecology and behavior [19].

**Figure 6:**
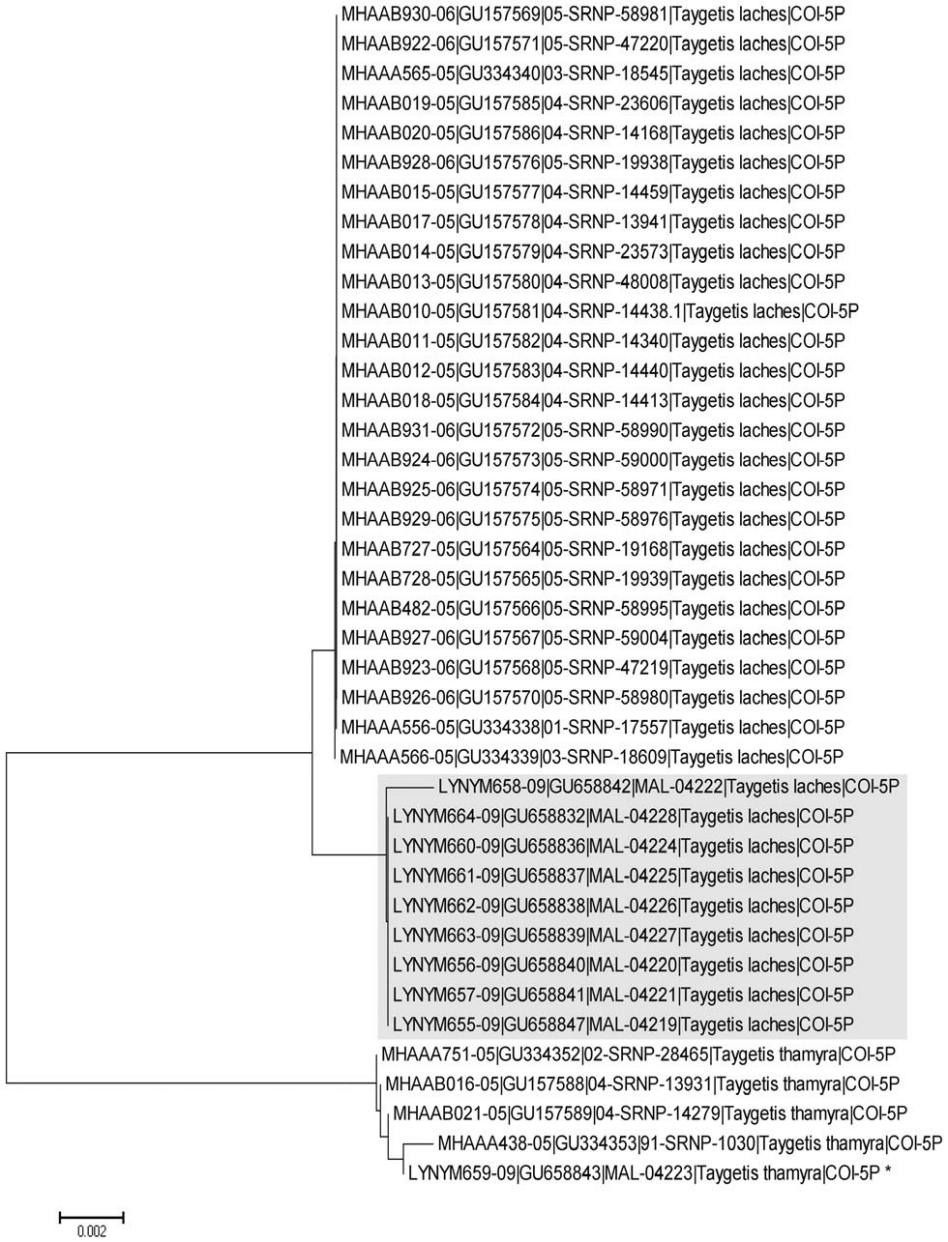
NJ tree showing specimens of *T. laches* and *T. thamyra* from Yucatan and Costa Rica. Specimens of *T. laches* (in grey) from the Yucatan group together and are differentiated from Costa Rican specimens, although they have less than 2% K2P divergence indicating they are likely geographic forms of a single species. The record marked with an asterisk derives from a specimen of *T. thamyra* from the Yucatan Peninsula which is grouped with Costa Rican specimens.

Specimens originally identified as *Taygetis thamyra* split into two well-defined clusters. The ID function in BOLD (Table S1) confirmed that one cluster was *T. thamyra* as it showed 100% of similarity to specimens from Costa Rica, while the second cluster showed 99.7% similarity with *Taygetis laches* from Costa Rica. Past research has shown difficulty in differentiating these two species and confusion by the use of the synonym *T. andromeda* instead *T. laches* [5], [34]–[36], but it was recently demonstrated [23] that *T. thamyra* and *T. laches* are well defined species. Figure 6 shows a NJ tree of Mexican and Costa Rican specimens for both species. Although Mexican and Costa Rican specimens of *T. laches* group together, the two clusters are distinct in the tree, indicating some phylogeographic structure. However, our results confirm the presence of *T. laches* from the Yucatan Peninsula and even from Mexico (Figure 1).

**Figure 7:**
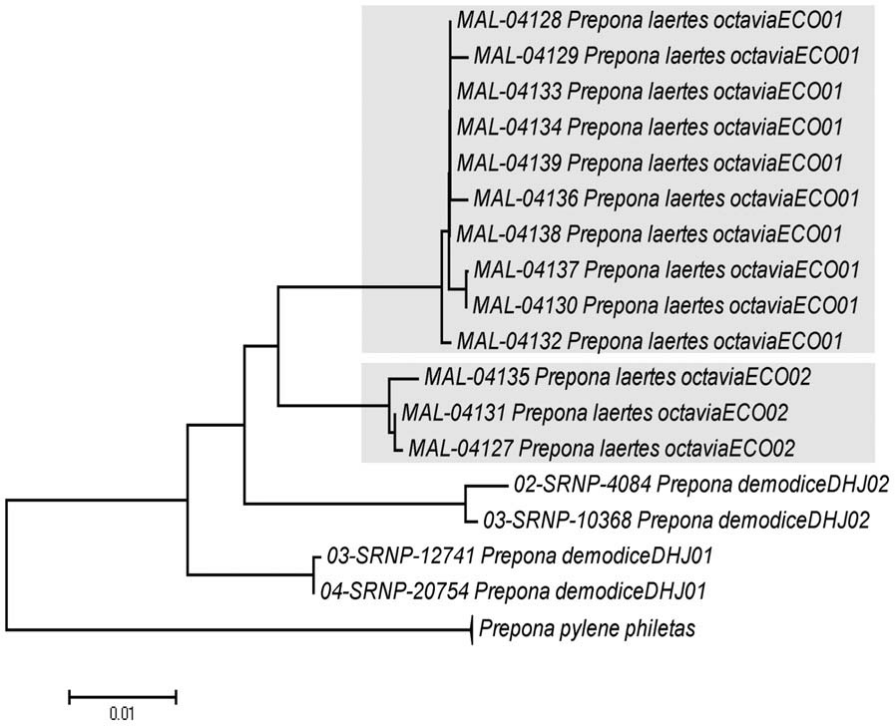
NJ tree showing species of *Prepona* from Yucatan Peninsula and Costa Rica. The two clusters of *P. laertes octavia* from the Yucatan are shown in grey. Note that similarly deep divergence was found in Costa Rican *P. demodice*. These two species were previously thought to be subspecies of *Prepona laertes* (*P. laertes octavia* and *P. laertes demodice*).

Subspecies are recognized for *Prepona laertes* (eg. *P. laertes octavia* and *P. laertes demodice*) [5], but Janzen [14], [23] has suggested that they deserve species status and we support this conclusion. Our *P. laertes* (Figure 3, H) is actually *P. octavia*, while the Costa Rican species is *P. demodice* [14]. Besides this, *Prepona* species show deep sequence divergence in Mexico and Costa Rica [23], forming two clusters for both species (Figure 7). Despite this fact, Mexican specimens are so morphologically similar to their Costa Rican counterparts that they cannot easily be distinguished, suggesting the need for a detailed morphological analysis. A recent barcode study on Mexican Preponini also noted the need for more detailed work on this group [37].

Seven other species were split in two clusters and one species was partitioned into three clusters that do not correspond to any known taxa. We ran the BOLD ID engine using the public database to seek matches with these splits (Table S1) and found that some of these species have similar splits in Costa Rica [11] (eg. *Adelpha basiloides* and *Biblis hyperia*, including three cluster in the latter species). Table 1 shows the species with splits and the matching taxa in the public species database on BOLD. The results also strongly suggest the presence of a cryptic species complex in *Hermeuptychia*, as this species splits in three clusters, but none matched with any records on BOLD (Figure 3, Table 1).

One of the clusters in three other species with splits, *A. idyja* (ECO01), *M. libye* (ECO02) and *P. laertes* (ECO01) matched with records for the same species in BOLD (Table 1). In the case of *A. idyja*, the subspecies *A. idyja argus* is reported from Mexico and a “melanic” form is recognized for this species [29]. Nevertheless, the deep divergence suggests that they are actually two species (Figure 3, B). Further work is needed to determine if this “unknown” cluster is actually the subspecies *A. idyja idyja* that has not previously been reported from Mexico as its distribution only includes Cuba, Isla de la Juventud, Hispaniola and Puerto Rico [38]. If so, both should be raised to a species level. As in this species, the other unknown clusters of *M. libye* (ECO01) and *P. laertes* (ECO02) need to be studied in detail, because current evidence indicates that they are also new species. In *Marpesia chiron* it is necessary to barcode more specimens as one cluster is only represented by one specimen and both clusters match with *Marpesia chiron* from BOLD (Table 1), and they not show any obvious morphological difference (Figure 3, G).

### Caterpillar identification

As in other studies that have used the BOLD ID engine to identify caterpillars [18], our analyses unambiguously assigned the 71 caterpillars that we analyzed to adults of 16 species of Nymphalidae analyzed in this study (Figures 2 and 4). Three specimens lacked a sequence record, but after the other larvae were identified by sequence analysis, it was possible to identify them by morphological comparisons.

Our work led to the first recognition of the caterpillar of the endemic species *Hamadryas julitta* (Figure 4, B) and revealed that it feeds on *Dalechampia schottii* (identified by images), a member of Euphorbiaceae. This host plant use is interesting because *H. glauconome* feeds on *D. scandens* in Costa Rica [11], [30] and both plant species occur in the Yucatan Peninsula, but *D. schottii* is endemic here [39], [40]. Nevertheless, more detailed study of food plant selection might help to provide insights into the factors driving speciation in the *H*. *julitta-glauconome* group.

### Final remarks

Extensive DNA barcode work has been carried out on several families of Lepidoptera in Costa Rica, and our study in the tropical southeast of Mexico complements this work, providing a comprehensive test of the efficacy of DNA barcoding for the most diverse of butterfly families, the Nymphalidae. Aside from confirming its effectiveness in species identification, we emphasize the utility of barcode analysis on the curation of natural history collections. Our work revealed a number of misidentified specimens and overlooked species in the ECOSUR collection. Our investigations also detected eight cases in which members of a species show substantial (>2%) sequence divergence, suggesting possible cases of cryptic species. Detailed analysis of external morphology, genitalia and ecological features are underway to determine if they are new species. We emphasize the need to analyze more specimens at least in those groups where there is either strong evidence or a suspicion of cryptic taxa. As well as its role in detecting overlooked species and misidentified specimens, DNA barcoding is a great asset in extending knowledge of life histories. The identification of immature stages is currently a very challenging task because there is no a key which provides the diagnostic characters to identify caterpillars to a species level. By contrast, we identified all nymphalid caterpillars that we examined to a species level by matching their barcode sequence to the reference library developed through our work on adults.

The present study highlights the value of constructing barcode reference libraries at regional levels as local factors drive the formation of endemic species. Mexico is such a heterogeneous landscape that it will not be surprising if future barcode studies discover many new cryptic species. We also emphasize the importance of the collective knowledge gained by combining results from different studies such as those carried out in Costa Rica and Mexico. Because access to such data enables morphological comparisons and aids rapid identifications, we conclude that is very important to move barcode records into open access as quickly as possible. This present study is just one component of a larger study on the Lepidoptera in Mexico that has already involved the sequence analysis of more than 7,000 larval and adult Lepidoptera. Subsequent studies will test the generality of results obtained in the present investigation.

## Materials and Methods

### Specimens

Over the past 20 years, staff, undergraduate and graduate students, and colleagues of El Colegio de la Frontera Sur-Chetumal (ECOSUR) have made extensive collections of Lepidoptera which are stored in the zoology museum under the acronym ECO-CH-L. Eight hundred and fifty-seven adults representing 121 morphologically identified species from this collection were analyzed in the present study. These specimens ranged in age from 1–30 years and all were collected from the Yucatan Peninsula including 169 from Yucatan State, 237 from Quintana Roo and 451 specimens from Campeche. Specimens were spread, photographed, labeled and morphologically examined to corroborate their identification. Morphological identifications were made through a) image comparison with specialized guides [e.g. 11,29,31,36,38]; b) specimen comparisons with other collections (e.g. Lepidoptera in the zoology museum of Facultad de Ciencias of Universidad Nacional Autónoma de México, UNAM); and c) for difficult groups we used specialized identification keys [eg. 30,33]. In addition, 74 nymphalid caterpillars collected on their host plant were photographed while alive whenever possible. These caterpillars were subsequently preserved in 96% ethanol and retained as vouchers in the Lepidoptera collection (ECO-CH-L). All specimen data including collection locality, GPS coordinates, date, collector, identifier and images are available in the project titled “Nymphalidae of the Yucatan Peninsula” in the Barcode of Life Data System (BOLD). All COI sequences have also been deposited in GenBank (http://www.ncbi.nlm.nih.gov/, see Table S2 for accession numbers).

We barcoded at least three adults of each species whenever possible. A 3 mm leg segment was removed from each adult, while our sampling protocol for caterpillars depended on their size. Three thoracic legs were removed from one side of the smallest larvae (2–5 mm length), while just one leg was removed from larger larvae. Each tissue sample was placed in a lysis plate well (96 wells Eppendorf® Plates) with a drop of 96% ethanol.

### Barcoding

Sequence analysis was carried at the Canadian Centre for DNA Barcoding following standard protocols [41]. DNA was extracted in 50 µl of lysis mix made of insect lysis buffer with Proteinase K and overnight digestion at 56°C. DNA isolation was done using a Glass Fiber plate (PALL2). The primers LepF (5’-ATTCAACCAATCATAAAGATATTGG-3’) and LepR (5’-TAAACTTCTGGATGTCCAAAAAATCA-3’) were used to PCR amplify a 658 bp fragment of COI. If these primers failed to generate an amplicon, another reverse primer EnhLepR (5’-CTCCWCCAGCAGGATCAAAA-3) was combined with LepF. Finally, a barcode record was recovered from a few of the oldest specimens by using two primer pairs that amplify short segments (307, 408 bp) of the barcode region -- MLepF (5’-GCTTTCCCACGAATAAATAATA-3’)-LepR and MLepR (5’-CCTGTTCCAGCTCCATTTTC-3’)-LepF.

Each PCR mix contained 6.25 µl of 10% trehalose, 2 µl of ddH_2_O, 1.25 µl of 10X buffer, 0.625 µl of 50 mM MgCl_2_, 0.125 µl of both primers (10 µM), 0.0625 µl of 10 mM dNTPs, 0.06 µl of Taq Polymerase and DNA template (2 µl). All PCR products were bidirectionally sequenced on an ABI 3730XL and reads were edited and assembled with Sequencher v. 4.8 (Gene Codes Corporation). Sequences and all collateral data from specimens are available on BOLD (www.boldsystems.org) in the project titled “Nymphalidae of the Yucatan Peninsula”.

### Sequences analysis

Sequence divergences were estimated using the Kimura two parameter (K2P) distance model [42]. The DNA-based identifications of specimens were validated whenever possible by examining sequence similarity with public records of other Nymphalidae available on BOLD (Table S1). Through MEGA 4.0 software [43], neighbor joining (NJ) analysis was used to gain a graphic representation of divergence values and caterpillar identification.

## Supporting Information

Table S1 **Accession codes from specimens of the public database, that match to some of the eight split species with interim name in this study.**
(XLS)

Table S2 **GenBank accession numbers for specimens in the “Nymphalidae of the Yucatan Peninsula” project.**
(XLS)
